# Comparisons of automated machine learning (AutoML) in predicting whistleblowing of academic dishonesty with demographic and theory of planned behavior

**DOI:** 10.1016/j.mex.2023.102364

**Published:** 2023-09-07

**Authors:** Rahayu Abdul Rahman, Suraya Masrom, Masurah Mohamad, Eka Nurmala Sari, Fitriani Saragih, Abdullah Sani Abd Rahman

**Affiliations:** a Faculty of Accountancy, Univesiti Teknologi MARA, Perak Branch, Tapah Campus, Malaysia; b College of Computing, Informatics and Mathematics, Universiti Teknologi MARA, Perak Branch, Tapah Campus, Malaysia; c Department of Accounting, Universitias Muhammadiyah Sumatera Utara, Indonesia; d Faculty of Science and Information Technology, Universiti Teknologi PETRONAS, Perak, Malaysia

**Keywords:** Whistleblowing, Academic dishonesty, AutoML, Demographic, Theory of planned behavior, *Automated Machine Learning (AutoML)*

## Abstract

Machine learning has been very promising in solving real problems, but the implementation involved difficulties mainly for the inexpert data scientists. Therefore, this paper presents an automated machine learning (AutoML) to simplify and accelerate the modeling tasks. Focused on Python and RapidMiner rapid modeling tools, Tree-based Pipeline Optimization Tool (TPOT) and AutoModel were used. This paper presents a comprehensive comparison between these tools with regard to the prediction accuracy and Area Under Curve (AUC) in classifying real cases of whistleblowing academic dishonesty among undergraduate students of two universities in Indonesia. Additionally, the correlations weight from demographic and Theory of Planned Behavior (TOB) attributes in the different machine learning models are also discussed. All the machine learning algorithms from TPOT and AutoModel are considerable powerful to generate good accuracy level (between 70–93% of AUC) in classifying both cases of whistleblowing and non-whistleblowing on the hold-out samples from the testing process. Generally, based on the validation results of the prediction models, demographic attributes presented more importance than the TBP attributes. The findings of this study will be a great interest of many research scholars to conduct a more in-depth analysis on AutoML for many domains mainly in education and academic misconduct fields.

• AutoML is the first of its kind to be empirically compared between TPOT and AutoModel in an application to predict academic dishonesty whistleblowing.
• Besides accuracy performances of the AutoML, the proportion of the variance of each attribute from demographic and Theory of Planned Behavior (TPB) is also presented in the prediction models of academic dishonesty whistleblowing.
• AutoML is a convenient and reproducible rapid modeling method of machine learning to be used in many kinds of prediction problem.

## Graphical abstract

**Figure:**
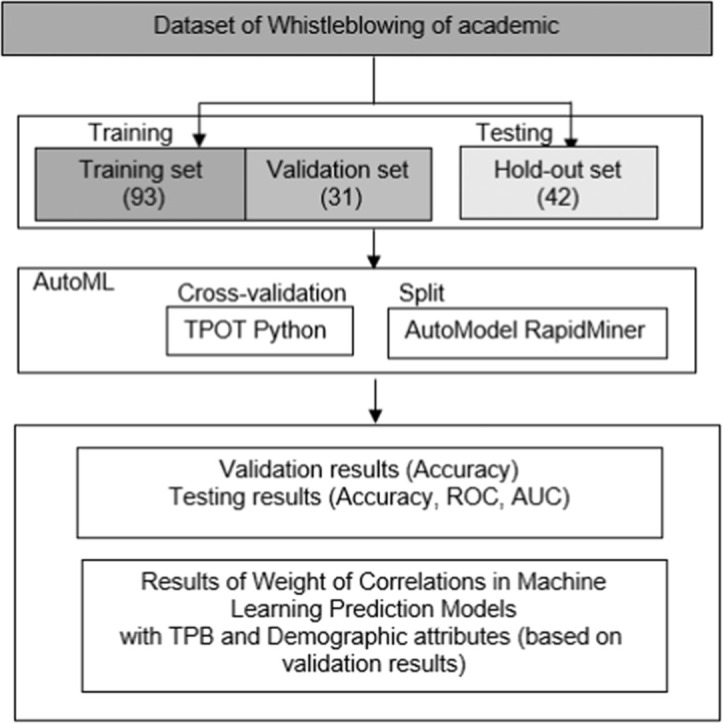

Specifications table

**Table:** 

Subject area:	Economics and Finance
More specific subject area:	*Education*
Name of your method:	*Automated Machine Learning (AutoML)*
Name and reference of original method:	Olson, R. S., & Moore, J. H. (2016, December). TPOT: A tree-based pipeline optimization tool for automating machine learning. In *Workshop on automatic machine learning* (pp. 66-74). PMLR.
Resource availability:	*TPOT is an open-source project on GitHub, and the underlying Python code can be found at*https://github.com/rhiever/tpot*.*
	*Python codes for this research are available at*https://colab.research.google.com/drive/1Jv9VimFfOUbD3Pw_8eZ0rsw27mEiENEB?usp=sharing
	*RapidMiner can be downloaded from*https://docs.rapidminer.com/9.9/studio/installation/.

## Introduction

Inexpert data scientists often deal with challenges to implement machine learning such as identifying the best features selection, hyper-parameter tuning, and handling imbalance dataset. Additionally, insufficient skills for computer programming and the complexity of extended codes scripts for machine learning implementation have given a significant hurdle for those data scientists. Decreasing the difficulties of the design and development for machine learning has become a major concern by many researchers recently due to the massive emergence of complex problems that need a more efficient and intelligent solutions. Machine learning is a kind of complex computing technique with artificial intelligence mechanisms that has the ability to infer new knowledge and redevelop learning like the way a human brain works [1]. Acknowledging the wider advantages of machine learning and the enormous demands from inexpert machine learning researchers, utilizing rapid and easy software tools is an important research issue in the implementation mainly for assisting the social sciences scholar from various domains, including in the education sector. Today, machine learning has been seen as a promising solution for teaching and learning problems, which has undergone assessment from the perspective of different contexts. To sustain the machine learning development and deployment in education, concentrating on the rapid and automated implementation will create new motivating and critical discussions around the educator policy makers.

One of the crucial problems in education that has received major concern is education dishonesty. Academic dishonesty can be defined as an intentional act of fraud [2], and a form of rule violation in higher institutions [3]. Examples of academic dishonesty includes plagiarism, assessment or examination cheating, illegal cooperation or collusion and ghost writing. In addition, researchers in [4] stress that a transition from face-to-face instruction to remote learning further intensifies the incident of academic dishonesty as well as the evolution of novel methods of cheating. In response, several initiatives have been implemented by the higher education institutions such as reformatting assessment using high-order thinking questions, code of ethics and integrity declaration prior examination and whistleblowing [5]. Whistleblowing is not new internal control mechanism, yet has been widely used by corporate sector in mitigating fraud by encouraging employees to report wrongdoing in organization. Nevertheless, the decision either to blow or not to blow the whistle (to report or not to report the misconduct) is a difficult and complex decision making as it exposes the whistleblower to several risks [6,7]. For an instance, in academic setting, whistleblowers face with social ostracism, name-calling and other forms of social sanctions from their academic peers [8]. Due to that, although a relatively large number of students are exposed to organization academic dishonesty, majority of them usually remain silent, which in turn lead the incidence of fraud as well as academic dishonesty is continuously high.

Despite the importance of whistleblowing in containing academic dishonesty, a review of literature on whistleblowing shows that most of prior studies more emphasize on whistleblowing practice in corporate setting [9,10]. Meanwhile, studies on classroom whistleblowing are very limited, and employed traditional statistical method in predicting whistleblowing intention [11], [12], [13], [14]. Understanding students’ attitude and perception toward whistleblowing will be useful for the university policymakers in prompting whistleblowing activities and establish whistleblower protection. Besides, an understanding of the factors that driving their intentions to whistleblowing should be useful to organizations that may employ these students upon graduation. Thus, this study aims to expand prior works that employed traditional statistical method with a new construct of students’ whistleblowing intention model on academic dishonesty using machine learning prediction technique based on automated machine learning (AutoML) method [15]. AutoML provides high degree of automation, including features or variables selection, machine learning algorithms selection and hyper-parameters optimization. For the non-expert data scientists, each of these steps may be challenging, resulting in significant hurdles to design and implement the algorithms. Thus, AutoML was introduced with aims to simplify the complex tasks for non-experts and to make it easier for them to use the techniques correctly and effectively. Besides, AutoML also provides additional benefits to the machine learning expert by accelerating the tedious steps.

This paper has two main contributions. First, it demonstrates the implementation of whistleblowing academic dishonesty prediction model using AutoML method to enable simple and efficient prediction analysis and to encourage the acceptance of whistleblowing as one of the universities mechanisms in mitigating academic misconducts. Second, it provides another design aspects of the machine learning whistleblowing prediction model by using a unified construct from the attributes of Theory of Planned Behaviour (TPB) as additional attributes of demographic. The influences of the attributes with regards to the performances of the machine learning models are also discussed in this paper. Therefore, this paper provides an important empirical contribution that fill the gap of research on whistleblowing academic dishonesty with the intelligent prediction approaches.

## Methods

### Sample of data

The dataset for this research was collected through survey questionnaires that personally disributed to undergraduate students of two universities in Indonesia. A total of 166 valid replies were used for the study out of the 292 questionnaires that were distributed, giving a response rate of 57 percent. The whistleblowing dataset from the questionnaire consists of students’ demographic information, such as academic grade (*Cumulative Grade Point Average* or *CGPA*), level of religiosity, integrity culture, course and their fear retaliation (the act or intention of hurting or doing something harmful to someone). Additionally, the data composed of factors that affecting such whistleblowing intention, which were constructed based on Theory of Planned Behavior (TPB). As listed in Table 1, the TPB as defined in [16,17] are composed of a set of attitudes (*affective* attitude and *instrumental* attitude), subjective norms toward the behavior (*injunctive norm* and *descriptive norm*), and perceptions of behaviour control (*self-efficacy* and *perceived controllability*).

**Table 1:** Descriptions of attributes of Theory of Planned Behavior (TPB).

Attribute	Indicators	Measurement	Eqs.
Attitude towards whistleblowing			
*Affective*	Pleasant, Enjoyable, Exciting	$\mathrm{Affective}=\frac{\sum_{k=1}^{3}{\mathrm{indicator}}_{k}}{3}$	Eq. (1)
*Instrumental*	Good, Beneficial, Valuable	$\mathrm{Instrumental}=\frac{\sum_{k=1}^{3}{\mathrm{indicator}}_{k}}{3}$	Eq. (2)
Subjective norm			
*Descriptive norm*	Academic misconduct is believed will be reported by i) Close people, ii) University people and iii) Friends	$\mathrm{Descriptive}=\frac{\sum_{k=1}^{2}{\mathrm{indicator}}_{k}}{2}$	Eq. (3)
*Injunctive norm*	Whistleblowing will be supported and approved by i) Close people, ii) University people and iii) Friends	$\mathrm{Injuctive}=\frac{\sum_{k=1}^{3}{\mathrm{indicator}}_{k}}{3}$	Eq. (4)
Behavior			
*Self-efficacy*	Ability, Confident, Easy to do	$\mathrm{Selfefficacy}=\frac{\sum_{k=1}^{3}{\mathrm{indicator}}_{k}}{3}$	Eq. (5)
*Perceived controllability*	Has resources, has opportunities, has total self-control	$\mathrm{Controllability}=\frac{\sum_{k=1}^{3}{\mathrm{indicator}}_{k}}{3}$	Eq. (6)

Attitudes refers to the degree to which a person has a favorable or unfavorable measure of the whistleblowing interest either through *affective* or *instrumental* attitudes measured with Eqs. (1) and (2) respectively. *Affective* attitude stresses more the emotional behavior aspects while instrumental factor in attitude emphasizes more the cognitive aspects of behavior.

Subjective norms towards the behavior refers to the belief on approving or disapproving. It relates to a person's principles about their peers or peoples surround either should engage in the behavior or not. A type of *subjective norms* is *descriptive norms* measured with Eq. (3), which is the perception of other peoples’ attitude towards whistleblowing. Another type is *injunctive norms* or social norms, calculated with Eq. (4) refers to the social support to perform the behavior.

Behavioral control or intention reflects to the motivational factors that control the behavior including *Self-efficacy* and *perceived controllability. Self-efficacy* as measured in Eq. (5) is defined as the student's confident to carry out the whistleblowing behaviour. The ability to control the whistleblowing behavior is defined as *perceived controllability* (Eq. (6)).

**Fig. 1:**
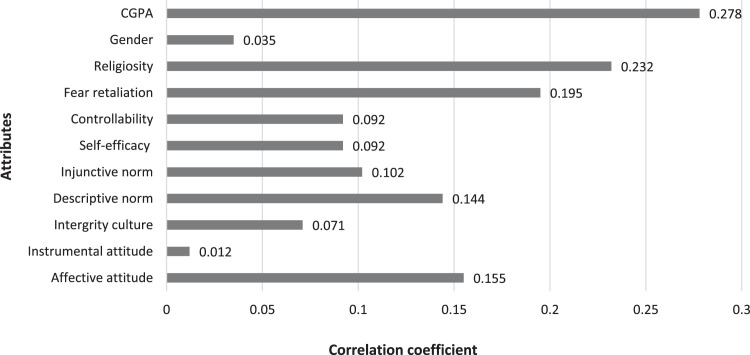
Correlation Coefficient of Whistleblowing Attributes.

The classification of whistleblowing is a type of binary classification in such that if the prediction probability value is 0.5 and above, the case is classify as 1 (whistleblowing). Otherwise, prediction value with less than 0.5 is resulted the model to classify the case as 0 (non-whistleblowing). On measuring the students’ whistleblowing intention to be used as the dependent variable (DV) of the prediction model, nine indicators have been used, including a general intention to whistleblowing and another whistleblowing measures on eight indicators of academic misconducts. Eq. (7) measure the value of whistleblowing intention.

(7) $$\mathrm{Whistleblowing}=\mathrm{general}+\;\frac{\sum_{k=1}^{8}{\mathrm{indicator}}_{k}}{8}$$

where general value can be either 1(yes) or 0 (no) to be added with the means of totals from the eight specific indicators. If the Whistleblowing above 2.5, the student whistleblowing intention was set to 1(whistleblowing). Before implementing the machine learning, correlations between all independent variables (attributes/features set) from demographic and TPB to the target variable (DV) were tested with Pearson Correlation technique, which depicted in Fig. 1.

Demographic attributes are CGPA, Gender, Religiosity, Fear Retaliation and Integrity culture while Controllability, Self-efficacy, Injunctive norm, Descriptive norm, Instrumental attitude and Affective attitude are the TPB attributes. The main problem appeared in the whistleblowing dataset is each of the attribute/feature has weak associations to the DV (below 0.3 correlation coefficients). With AutoML that able to optimize the best combination of features, the following research questions have been constructed to be justified from the results generated in each of the machine learning algorithm.

RQ1: Does each attribute is being utilized in all the machine learning algorithms suggested by AutoML?
RQ2: How variances of importance can be presented by each of the attribute in the different machine learning algorithms?

To elaborate the answer for RQ1, it is useful to get insight on the implication of weight of correlations to the algorithms’ performances. Moreover, by observing the variances of important to answer the RQ2, different effect between Demographic and TPB can be generally described. Academic performance (*CGPA*) has the strongest correlation to the whistleblowing intention in Fig. 1 but does it remain important in AutoML is another point to be elaborated for answering the RQ2.

### Automated machine learning (AutoML)

Most AutoML use optimization search to select the best machine learning pipelines. The early work on AutoML used Bayesian optimization [18] to be supported in software tools like Auto-Weka [19,20] and Auto-SkLearn [21]. TPOT [22] and AutoModel in RapidMiner [23] are the recent technology for automated machine learning. In [15], the researchers introduced PVPF tool to implement automated machine learning suitable for photovoltaic power forecasting based on Bayesian Regulation algorithm to neural networks machine learning. Focusing on health domain, researchers have highlighted the crucial need to use automated machine learning in healthcare based on a wide array of literature of automated machine learning [18]. From the study, the researchers suggested some useful automated machine learning tools for the inexpert data scientists including Google Cloud's AutoML system, Amazon SageMaker, Driverless AI, Microsoft Azure AutoML and DataRobot.

This paper presents two software platforms that have ability to support AutoML namely Python and RapidMiner. In Python, there are number of libraries that support AutoML but the recent interesting project is provided by the TPOT library [20,^24^,25]. TPOT uses Genetic Programming [25] for optimizing the best machine learning models. RapidMiner provides AutoModel based on Grid Search optimization [26] to execute the automation of machine learning modeling. Pipeline optimizations and hyper-parameters optimization are the main benefits of automated machine learning. Like TPOT and AutoModel, meta-heuristics algorithm is the technique for optimizing the machine learning pipelines. Machine learning pipelines are the collection of output from a series of machine learning processes started from data exploration and pre-processing, features engineering, algorithm selection, hyper-parameters configuration and tuning. Different with manual machine learning that manually executed by human, there are a number of computing mechanisms in automated machine learning that used search optimization for automating the processes mainly in selecting features of the models, identifying the suitable algorithms and hyper-parameters tuning [27,28].

Python's TPOT was chosen for its ability to automate the end-to-end machine learning process, providing various model selection strategies, pre-processing techniques, and automated hyper-parameters tuning. The complete Python codes to execute all the machine learning process is very short to be replicated. RapidMiner's AutoModel was selected due to its intuitive graphical interface that caters to users with varying levels of expertise, allowing inexpert data scientists to utilize AutoML tools effectively. Most importantly, both TPOT and RapidMiner have garnered popularity in the machine learning community. Extensive libraries are available for these rapid tools along with the strong support community, allowing more advanced data analytics.

Additionally, the ability of these rapid tools to mitigate the impact of class imbalance while identifying optimal models further strengthens their suitability for this study. TPOT can effectively address the challenges posed by the imbalanced dataset by employing oversampling and under sampling techniques during the optimization process. AutoModel demonstrated its capability in handling imbalanced datasets by utilizing ensemble-based algorithms and specialized techniques. The tool employed strategies like cost-sensitive learning and ensemble pruning to ensure that the final selected models were not biased towards the majority class. Although the target label used in this study exhibits more than just class imbalance (with 57.23% falling within range 2 and 42.77% within range 1), it's important to note the presence of imbalanced features within the dataset. Both TPOT and AutoModel incorporate algorithmic and ensemble approaches that take into account not only the distribution of the target label but also the potential consequences of skewed feature representations.

*TPOT in Python*

**Fig. 2:**
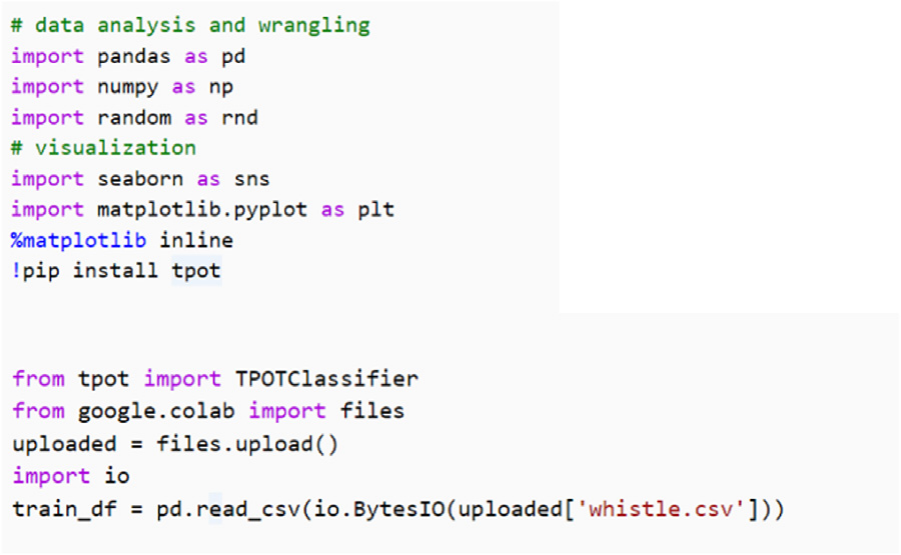
Python codes to install TPOT and read dataset.

**Fig. 3:**
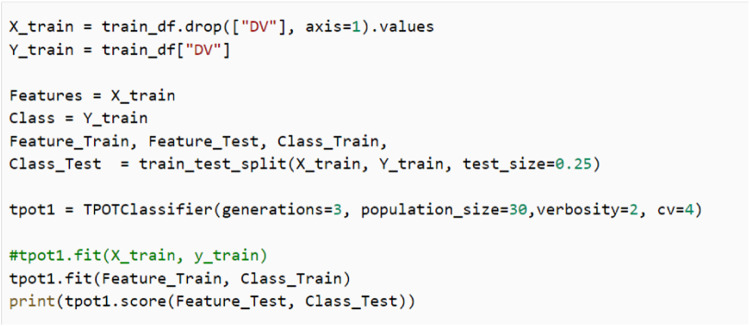
Python codes to implement TPOT.

TPOT library can be imported in any programming files for developing the machine learning that can be written in any Python development editor. The basic codes for TPOT library in Python codes written with Google Colab, a web browser for Python development editor. The main part to install TPOT library and to read the dataset is given in Fig. 2. Then, to implement the training and prediction, Python codes as in Fig. 3 can be used.

**Fig. 4:**
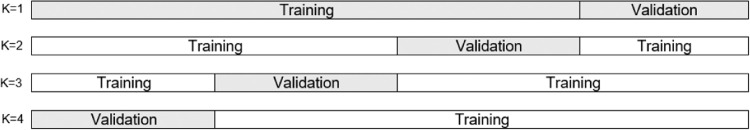
Cross-Validation with 4 K-Folds (Training=93, Validation=31).

TPOT uses Cross-Validation (CV) training approach that has been set with 4 K-Folds and the test size is 0.25 representing 42 numbers out of 166 data were used for testing and the rest 124 for training and validation (Refer Fig. 4). From the 124 data, 91 were used for training and 31 for validation. K-Fold CV is a resampling procedure used to ensure that the machine learning models can perform better than the simple split training approach. The K is referred as the number of groups that a given sample data can be divided.

Generation is the number of iterations for the optimization search while population is the number of maximum individual (pipelines) to be randomly selected in the optimization search. The findings in [29] suggested that default configuration by TPOT itself was able to generate good prediction results when tested on some common benchmark problems used in the study. The higher of population numbers is expected to provide more probability for the TPOT to select achieve better optimal models but it is depending the tested dataset, which should be observed in this research.

### AutoModel in RapidMiner

**Fig. 5:**
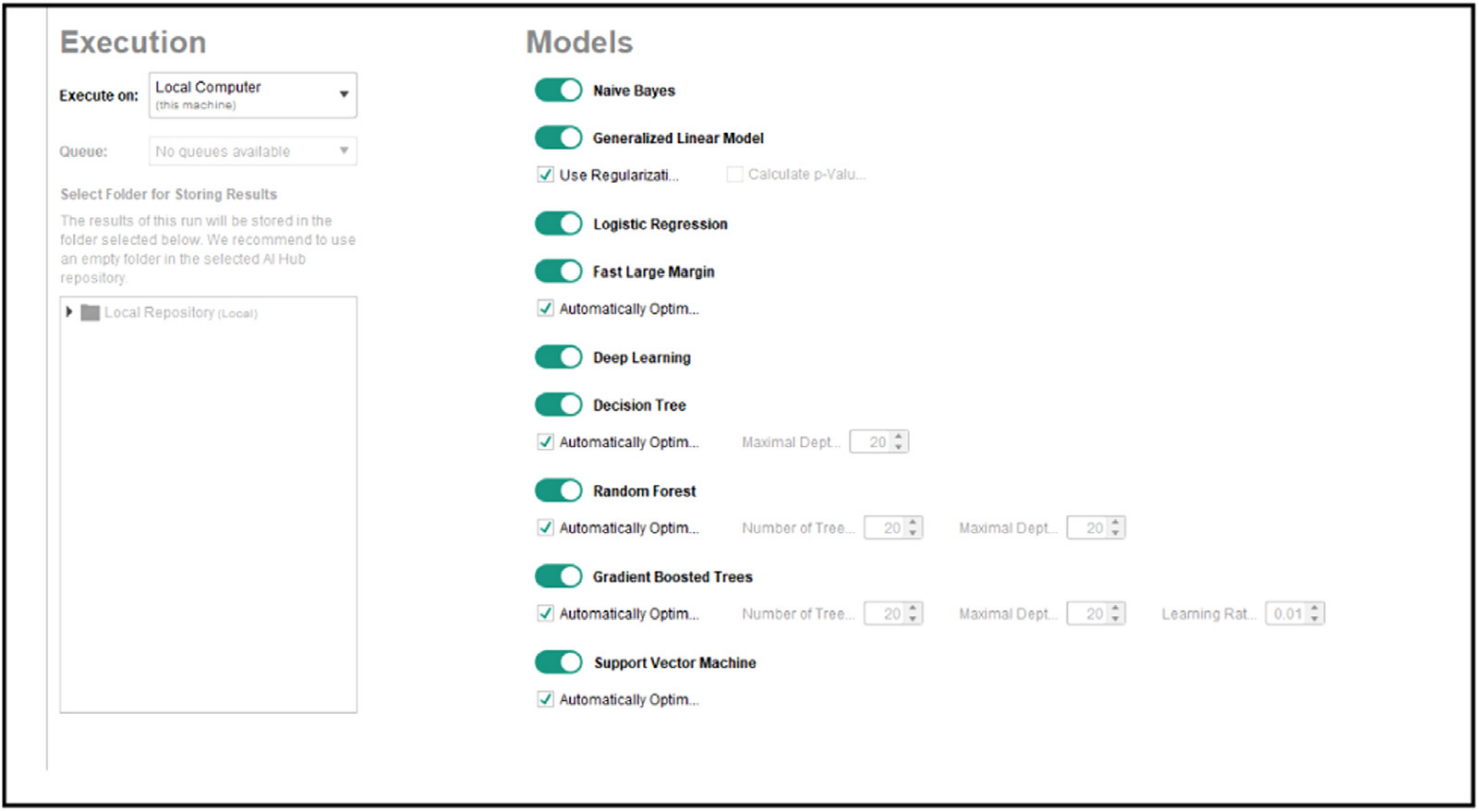
Suggested Machine Learning Algorithms in AutoModel.

**Fig. 6:**
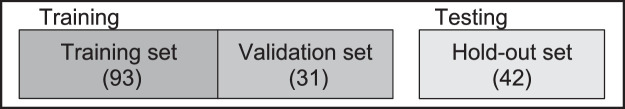
Training, validation and testing dataset.

**Fig. 7:**
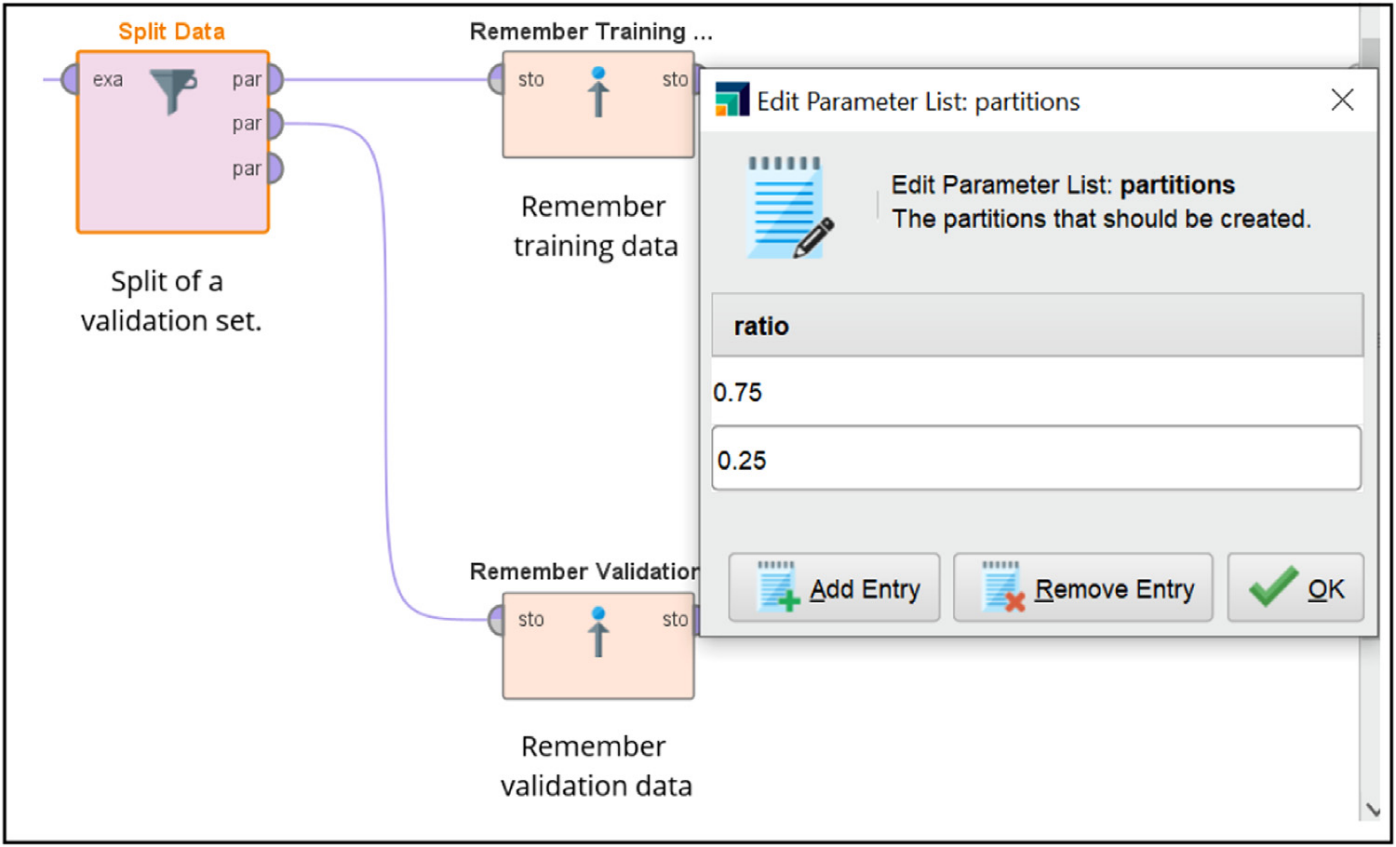
Split-Validation in AutoModel.

AutoModel in RapidMiner with Graphical User Interface (GUI) is easier than TPOT that used Python programming. Based on the whistleblowing dataset, AutoModel suggested a series of machine learning to be observed. As seen in Fig. 5, AutoModel suggested nine suitable algorithms for the whistleblowing dataset but only the tree best outperformed algorithms were selected in this research namely Naïve Bayes, Generalized Linear Model and Logistic Regression. For each algorithm, AutoModel used split validation approach hence the performances results were generated based on the 31 number of the validation dataset. Thus, 93 data were used for training set and the rest 41 hold-out data is for testing as illustrated in Fig. 6. The GUI to set the ratio of data from the training dataset is given in Fig. 7. Testing the selected machine learning models were executed in different process of model deployment to read the testing data and did the prediction.

### Performance metrics

The evaluation of prediction accuracy in this study is emphasized by the most common metrics of accuracy and classification error. However, these metrics have a limitation as they assess the prediction model's performance without distinguishing its performance with respect to specific classes. Since the machine learning models in this research address a binary classification problem involving whistleblowing and non-whistleblowing students, it becomes essential to gauge the machine learning ability to accurately predict each class. Moreover, this becomes particularly crucial with the inherent imbalance in the target label distribution of the dataset. To address this, sensitivity (True Positive Rate) and specificity (False Positive Rate) were employed as supplementary metrics. Sensitivity denotes the total number of correct predictions for the whistleblowing cases, while specificity signifies the accurate prediction of non-whistleblowing cases. To provide a more comprehensive visualization of the trade-off between sensitivity and specificity, this study employed a Receiver Operating Characteristics (ROC) Curve graph. The Area Under Curve (AUC) of the ROC curve was then utilized to quantify the model's overall classification ability. A higher AUC indicates a more potent model in effectively classifying both cases of whistleblowing and non-whistleblowing students. By utilizing these metrics and techniques, the research design gains robustness through a clearer description of the model's performance with respect to the specific challenges posed by the imbalanced distribution of the target label.

### Validation and testing

Table 2 presents the output from TPOT Python that suggested different machine learning algorithms with the optimal hyper-parameters from different run of population sizes. Validation accuracy at each iteration/generation from the validation process was printed out to be averagely calculated. The testing accuracy is displayed from the statement print (tpot.score (Feature_Test,Class_Test)). In average, all algorithms suggested by TPOT able to achieve good validation and testing accuracies and the most outperformed is the GradientBoostingClassifier when the population size was set to 40.

**Table 2:** Results of prediction from TPOT Python.

Population size	Suggested machine learning algorithm with optimal hyper-parameters	Validation accuracy	Testing accuracy
10	RandomForestClassifier(MLPClassifier(input_matrix, alpha=0.001, learning_rate_init=0.1), bootstrap=False, criterion=gini, max_features=0.2, min_samples_leaf=8, min_samples_split=4, n_estimators=100)	0.67	0.69
20	GaussianNB (Nystroem(input_matrix, gamma=0.30000000000000004, kernel=cosine, n_components=8))	0.69	0.60
30	BernoulliNB(XGBClassifier(input_matrix, learning_rate=0.5, max_depth=3, min_child_weight=8, n_estimators=100, n_jobs=1, subsample=1.0, verbosity=0), alpha=10.0, fit_prior=False)	0.71	0.55
40	GradientBoostingClassifier(CombineDFs(input_matrix, SGDClassifier(input_matrix, alpha=0.001, eta0=0.1, fit_intercept=True, l1_ratio=0.5, learning_rate=constant, loss=log, penalty=elasticnet, power_t=0.5)), learning_rate=0.01, max_depth=7, max_features=0.9000000000000001, min_samples_leaf=19, min_samples_split=9, n_estimators=100, subsample=0.6000000000000001)	0.72	0.66

**Fig. 8:**
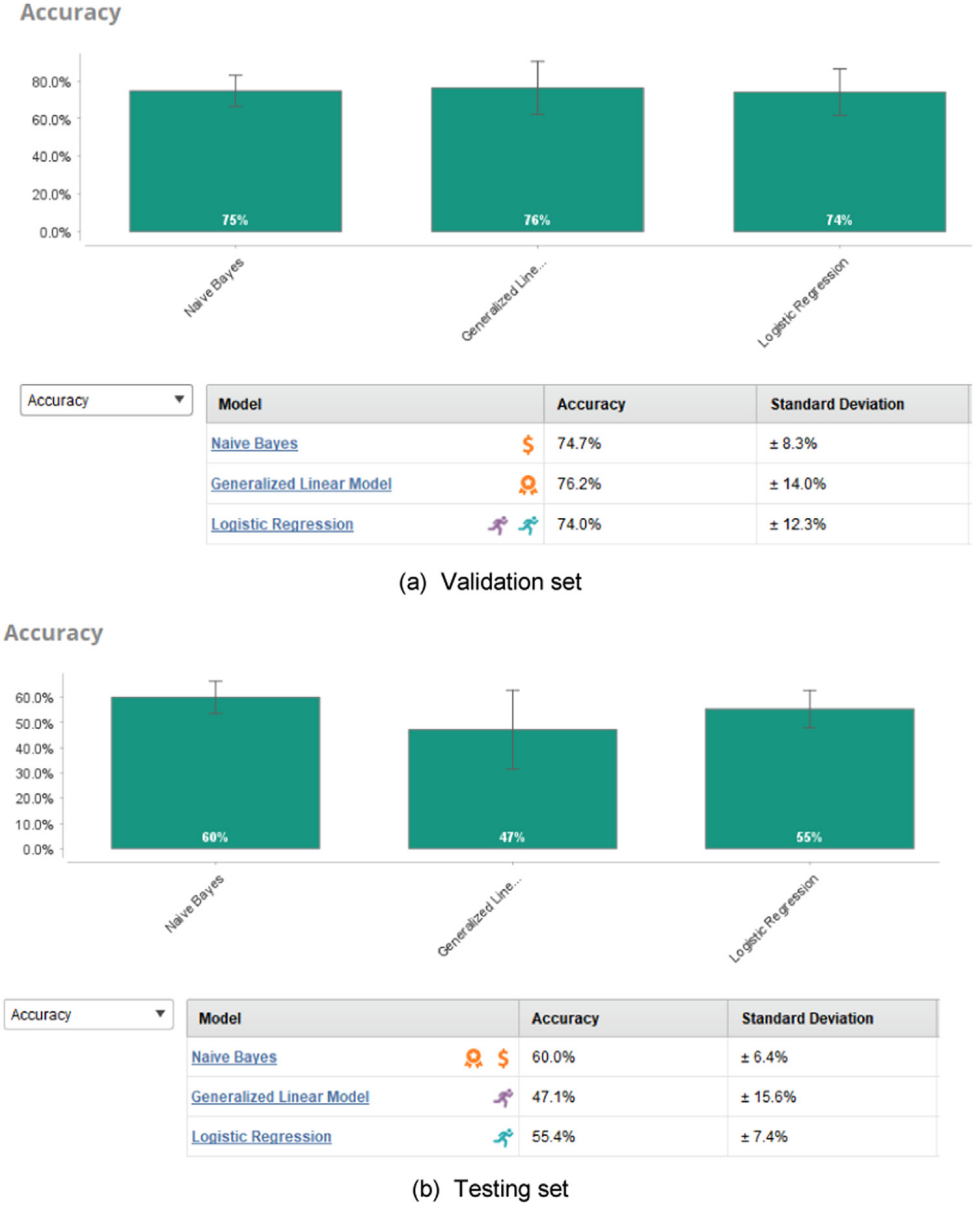
The accuracy output form AutoModel validation and testing.

Furthermore, Fig. 8 showed the results provided by AutoModel in RapidMiner platform from validation and testing phases. All the three algorithms (Naïve Bayes, Generalized Linear Model and Logistic Regresson) suggested by AutoModel have generated higher accuracy than TPOT from the validation set but has lower ability (between 60–55%) than TPOT when tested on the hold-out samples during the testing. Although TPOT seems more complicated with the Python codes than AutoModel that used GUI, TPOT can support fast implementation with one time execution (run) to get the validation and testing results. However, the Python programs for TPOT are easily replicated by researchers with the very short lines of codes. Once the knowledges and skills have been grasps, a variety of machine learning prediction models can be run easily for many applications. Compared to AutoModel, two different processes or project have to be developed in RapidMiner, which can be time consuming and wasting of the computer resources.

**Fig. 9:**
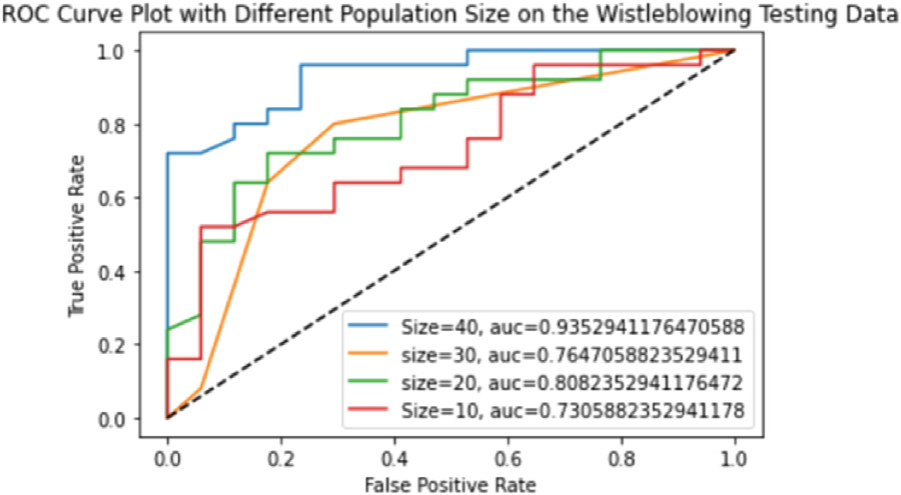
TPOT ROC and AUC.

**Fig. 10:**
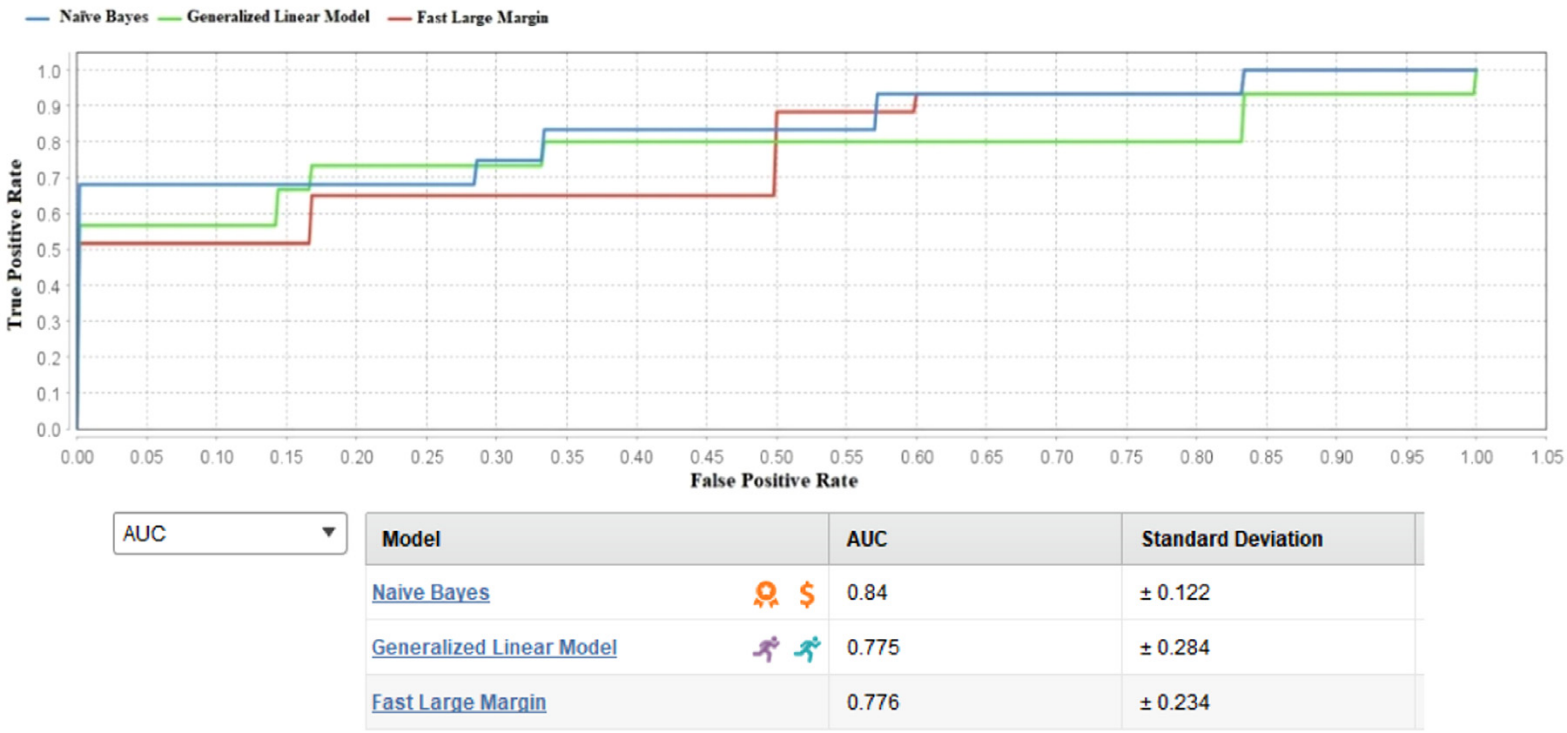
AutoModel ROC and AUC.

The following findings are described based on the ROC and AUC of the AutoML. Fig. 9 presents the ROC and the AUC of TPOT based on the testing data at different number of populations. As expected, the higher population numbers increased the AUC results in consistent with the accuracy results in such that better accuracy can be achieved with higher number of populations. The theory of genetic and evolutionary algorithms has discovered that long processing time will happen with better accuracy in accordance to the higher number of populations [30]. The TPOT and AUC from AutoModel on the tested data are depicted in Fig. 10. It can be seen that the Naïve Bayes was outperformed other algorithms in the AutoModel and but has less AUC value than TPOT. In general, all the machine learning algorithms from TPOT and AutoModel are considerable powerful to generate higher accuracy in classifying both cases of whistleblowing and non-whistleblowing on the hold-out samples from the testing process.

### Weight of correlations in the AutoML

Furthermore, it is important in this research to understand how each of whistleblowing attributes from the demographic and TPB effect the results of the different machine learning models. These can be interpreted from the weight of correlations of each attribute in all the machine learning models. Table 3 lists the attributes’ weight of correlations in the different machine learning algorithms from the validation dataset.

**Table 3:** Attributes’ weight of correlations in the AutoML.

Attribute	TPOT	AutoModel		
	GradientBoostingClassifier	Naïve Bayes	Generalized Linear Model	Fast Large Margin
TPB				
Affective attitude	0.022	0.039	0.049	0.047
Instrumental attitude	0.023	**0.218**	0.206	0.182
Descriptive norm	0.079	0.047	0.014	0.065
Injunctive norm	0.001	0.045	0.032	0.179
Self-efficacy	0.023	0.084	0.198	0.208
Controllability	0.025	0.050	0.051	0.138
Demographic				
Gender	0.009	0.024	0.028	0.043
CGPA	0.042	0.157	**0.320**	**0.223**
Religiosity	**0.175**	0.048	0.052	0.080
Fear retaliation	0.124	0.034	0.049	0.073
Integrity culture	0.014	0.078	0.058	0.068

Table 2 shows that all the attributes are contributed at some level of knowledge to each of the machine learning algorithm although most of them have very low weight of correlations. This finding is the answer for the RQ1. All the attributes in GradientBoostingClassifier by TPOT present lower weight of correlations resulted its lower performance compared to the three machine learning algorithms in AutoModel RapidMiner, (based on the validation set).

Furthermore, in order to provide a rationale for addressing Research Question 2, the information presented in Table 2 indicates that demographic characteristics have emerged as the primary influencing factor across the majority of machine learning algorithms, as compared to attributes based on the Theory of Planned Behavior (TPB). Notably, the Generalized Linear Model assigns a weight of 0.32 to CGPA, while the Fast Large Margin attributes a weight of 0.223 to this academic achievement indicator.

These findings emphasize the significance of CGPA not only within various machine learning algorithms but also outside the model contexts (Refer to Fig. 1). The positive correlation revealed by the Pearson test in Fig. 1 supports the idea that higher academic achievement correlates with an increased likelihood of whistleblowing among students. This observation aligns with similar conclusions drawn in [31]. Furthermore, Religiosity, another demographic attribute has emerged as the most influential factor in the Gradient Boosting Classifier, which indicates that a stronger religious inclination corresponds to a heightened sense of responsibility towards reporting academic misconduct, as noted in [32]. CGPA is the third importance feature in Gradient Boosting Classifier and is the second in Naïve Bayes, indicating the academic achievement as substantial significance.

Compared to demographic attributes, every TPB attribute demonstrates a diminished impact on all machine learning algorithms, with the exception of the Instrumental attribute that emerges as the primary influencer in the Naive Bayes algorithm. The results of this reduced influence are in accordance with the observations presented in Fig. 1, as indicated by the Pearson test.

The performance of different machine learning algorithms can be explained by the findings of this weight correlation analysis. Within the Generalized Linear Model and Fast Large Margin algorithms, the attribute with the highest influence is CGPA, leading to AUC scores of 0.78 for both algorithms. This underlines that CGPA's significant predictive power in discerning the behavior under consideration within these models.

Notably, the attribute of religiosity emerges as the most crucial factor within the algorithm of Gradient Boosting Classifier, resulting in an impressive AUC score of 0.9. This highlights the essential role of religious inclination in predicting and explaining certain behaviors within the context of this algorithm.

In the Naive Bayes algorithm, the most influential attribute stems from the TPB framework, which is the instrumental attitude. This attribute is closely followed by CGPA, which collectively contributes to an AUC score of 0.84. This signifies that while religiosity and academic achievement play pivotal roles in other algorithms, the Naive Bayes algorithm places emphasis on the role of instrumental attitude from TPB in making accurate predictions.

This paper presents a comprehensive comparison between software tools that support AutoML. With the utilization of real data in this study, a critical challenge to implement machine learning is the data had very weak connections between its variables. This problem made it hard for the conventional prediction model to give really accurate results and lengthy time is involved for the inexpert data scientists to conduct hyper-parameters tuning. Thus, the introduction of AutoML has become advantageous as it automates the identification of the optimal variable combinations for enhancing model performance. Within the tested dataset, TPOT is proven to have better performances than AutoModel in term of accuracy and AUC but the implementation of TPOT is more difficult than AutoModel. Nevertheless, the Python codes for TPOT are very easy to be replicated by researchers. Additionally, this paper presents comparison for variance of contributions between the attributes that ware constructed for the whistleblowing academic dishonesty classification model based on demographic and TPB theory. The findings reveal the intricate interplay between attributes and algorithms in machine learning. The algorithm-specific importance of certain attributes and emphasizing the need to consider different attributes depending on the chosen algorithm is demonstrated from the findings. The strong association between religiosity, CGPA, and instrumental attitude in their respective algorithms showcases the nuanced nature of predictive factors and their impact on model performance. Further research could investigate deeper into the underlying reasons for these attribute-algorithm associations and potentially yield insights into the psychological and behavioral dynamics they represent.

## Ethics statements

Participants are aware of the specific privacy statements and confidentiality protections in online surveys. Participants' consent was collected through a Google form, and the data has been completely anonymised.

## CRediT authorship contribution statement

**Rahayu Abdul Rahman:** Conceptualization, Methodology, Formal analysis, Data curation. **Suraya Masrom:** Writing – original draft, Formal analysis, Data curation. **Masurah Mohamad:** Conceptualization, Methodology, Writing – review & editing. **Eka Nurmala Sari:** Data curation. **Fitriani Saragih:** Data curation. **Abdullah Sani Abd Rahman:** Conceptualization, Methodology.

## Declaration of Competing Interest

The authors declare that they have no known competing financial interests or personal relationships that could have appeared to influence the work reported in this paper.

## Data Availability

Data will be made available on request.

## References

[1] Salkuti S.R. (2020). A survey of big data and machine learning. Int. J. Electr. Comput. Eng..

[2] Achmada T., Ghozali I., Pamungkas I.D., Nuswantoro U.D. (2020). Detection of academic dishonesty: a perspective of the fraud pentagon model. Int. J. Innov. Creat. Change.

[3] Dendir S., Maxwell R.S. (2020). Cheating in online courses: evidence from online proctoring. Comput. Hum. Behav. Rep..

[4] Peled Y., Eshet Y., Barczyk C., Grinautski K. (2019). Predictors of academic dishonesty among undergraduate students in online and face-to-face courses. Comput. Educ..

[5] Elsalem L., Al-Azzam N., Jum'ah A.A., Obeidat N. (2021). Remote E-exams during COVID-19 pandemic: a cross-sectional study of students’ preferences and academic dishonesty in faculties of medical sciences. Ann. Med. Surg..

[6] Xiao F., Wong-On-Wing B. (2021). Employee sensitivity to the risk of whistleblowing via social media: the role of social media strategy and policy. J. Bus. Ethics.

[7] Iwai T., Yeung L., Artes R. (2021). Voice or silence: antecedents of whistleblowing intentions. RAUSP Manag. J..

[8] Khan J. (2022). Examining whistleblowing intention: the influence of rationalization on wrongdoing and threat of retaliation. Int. J. Environ. Res. Public Health.

[9] Lee G., Xiao X. (2018). Whistleblowing on accountingy-related misconduct: a synthesis of the literature. J. Acc. Lit..

[10] Brink A.G., Lowe D.J., Victoravich L.M. (2017). The public company whistleblowing environment: perceptions of a wrongful act and monetary attitude. Acc. Public Interest.

[11] Alshurideh M., Al Kurdi B., Salloum S.A., Arpaci I., Al-Emran M. (2020). Predicting the actual use of m-learning systems: a comparative approach using PLS-SEM and machine learning algorithms. Interact. Learn. Environ..

[12] Priyadarshini A., Mishra S., Mishra D.P., Salkuti S.R., Mohanty R. (Sep. 2021). Fraudulent credit card transaction detection using soft computing techniques. Indones. J. Electr. Eng. Comput. Sci..

[13] Suparwito H., Polina A.M., Budiraharjo M. (2021). Student perceptions analysis of online learning: a machine learning approach, Indones. J. Inf. Syst..

[14] Salih N.Z., Khalaf W. (Sep. 2021). Prediction of student's performance through educational data mining techniques. Indones. J. Electr. Eng. Comput. Sci..

[15] Alomari M.H., Adeeb J., Younis O. (2019). PVPF tool: an automated web application for real-time photovoltaic power forecasting. Int. J. Electr. Comput. Eng..

[16] Ajzen I. (2020). The theory of planned behavior: frequently asked questions. Hum. Behav. Emerg. Technol..

[17] Dunn R., Hattie J., Bowles T. (2018). Using the theory of planned behavior to explore teachers’ intentions to engage in ongoing teacher professional learning. Stud. Educ. Eval..

[18] Waring J., Lindvall C., Umeton R. (January 2020). Automated machine learning: review of the state-of-the-art and opportunities for healthcare. Artif. Intell. Med..

[19] Kotthoff L., Thornton C., Hoos H.H., Hutter F., Leyton-Brown K. (2017). Auto-WEKA 2.0: automatic model selection and hyperparameter optimization in WEKA. J. Mach. Learn. Res..

[20] C. Thornton, F. Hutter, H.H. Hoos, and K. Leyton-Brown, “Auto-WEKA: Combined selection and hyperparameter optimization of classification algorithms,” in Proceedings of the 19th ACM SIGKDD International Conference on Knowledge Discovery and Data Mining, 2013, pp. 847–855.

[21] Feurer M., Eggensperger K., Falkner S., Lindauer M., Hutter F. (2022). Auto-sklearn 2.0: hands-free automl via meta-learning. J. Mach. Learn. Res..

[22] Olson R.S., Moore J.H. (2019). Automated Machine Learning: Methods, Systems, Challenges.

[23] M. Bjaoui, H. Sakly, M. Said, N. Kraiem, and M.S. Bouhlel, “Depth insight for data scientist with RapidMiner an innovative tool for AI and big data towards medical applications{\guillemotright},” in Proceedings of the 2nd International Conference on Digital Tools & Uses Congress, 2020, pp. 1–6.

[24] R.S. Olson and J.H. Moore, “TPOT: a tree-based pipeline optimization tool for automating machine learning,” in Proceedings of the Workshop on Automatic Machine Learning, 2016, vol. 64, pp. 66–74. [Online]. Available: https://proceedings.mlr.press/v64/olson_tpot_2016.html

[25] Langdon W.B., Harman M. (Sep. 2015). Optimizing existing software with genetic programming. IEEE Trans. Evol. Comput..

[26] H. Alibrahim and S.A. Ludwig, “Hyperparameter optimization: comparing genetic algorithm against grid search and bayesian optimization,” in Proceedings of the IEEE Congress on Evolutionary Computation (CEC), 2021, pp. 1551–1559.

[27] Slimani I. (2022). Automated machine learning: the new data science challenge. Int. J. Electr. Comput. Eng..

[28] Poolwan J., Smanchat S. (2018). An architecture for simplified and automated machine learning. Int. J. Electr. Comput. Eng..

[29] Masrom S., Mohamad M., Hatim S.M., Baharun N., Omar N., Rahman A.S.A. (2020). Different mutation and crossover set of genetic programming in an automated machine learning. IAES Int. J. Artif. Intell..

[30] Nayyar A., Garg S., Gupta D., Khanna A. (2018). Advances in Swarm Intelligence for Optimizing Problems in Computer Science.

[31] Bernawati Y., Saputra R.S. (2020). The effect of individual factors, subjective norms, and self-efficacy on the intention of whistleblowing: a case of students of the faculty of economics and business, Airlangga University. Public Manag. Account. Rev..

[32] Jullum M., Løland A., Huseby R.B., Ånonsen G., Lorentzen J. (2020). Detecting money laundering transactions with machine learning. J. Money Laund. Control.

